# Utility of CMR for differentiating acute from chronic myocardial infarction - Revisiting T2-weighted imaging with inclusion of intermediate aged infarcts

**DOI:** 10.1186/1532-429X-13-S1-O68

**Published:** 2011-02-02

**Authors:** Martijn Smulders, Sebastiaan Bekkers, Han Kim, Michele Parker, Raymond Kim

**Affiliations:** 1 Maastricht University Medical Center, Maastricht, Netherlands; 2 Duke University Medical Center, Durham, NC, USA

## Introduction

Published reports have concluded that T2-weighted CMR (T2W-CMR) is highly accurate in differentiating acute from chronic MI. However, the majority of patients investigated had infarcts <1-week-old or >6-months-old. Clinically, it would be vital to distinguish an infarct a few days versus a few months old, however some studies suggest T2W-CMR edema may persist for months [Heart 2001;85:639-42, AHJ 2007;154:929-36], possibly precluding this differentiation.

## Purpose

The study primary aim was to assess the prevalence of T2W-CMR edema across a range of infarct ages and to assess its accuracy with and without the inclusion of intermediate-aged infarcts. Secondary aims were to evaluate other CMR markers of acute MI, and to compare image quality of CMR techniques.

## Methods

221 CMR studies were performed at various time points post-MI in 117 first ST-elevation-MI patients enrolled prospectively and consecutively from two CMR centers. Prespecified markers of "acute" MI were hyperintensity on T2W-CMR, microvascular obstruction (MO) on delayed-enhancement-CMR (DE-CMR), and increased end-diastolic wall thickness (Increased-EDWT, >150% of remote measured quantitatively) on cine-CMR. Images were scored blinded to identity and all clinical information. Individual CMR techniques were interpreted separately. Image quality and frequency/severity of artifacts were also evaluated.

## Results

**Figure 1:**
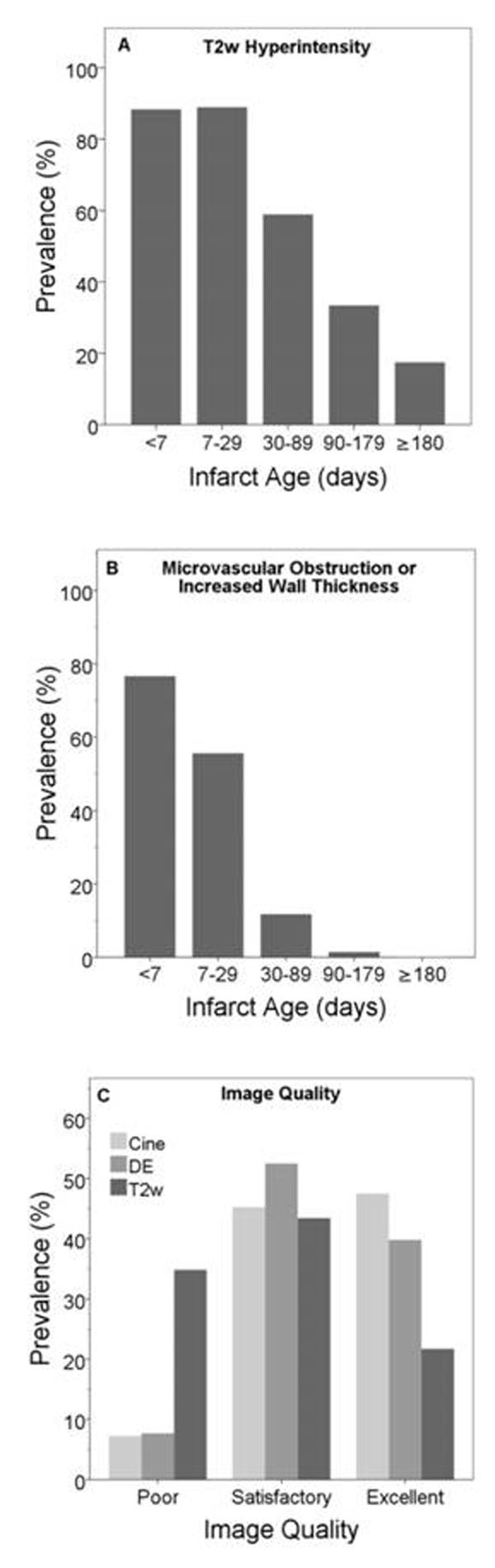


Mean age was 58±11 years; 84% were men. Prevalence of T2W-CMR hyperintensity steadily decreased for older infarcts starting 1-month post-MI but was still substantial for 1-6 month-old infarcts (Figure 1, Panel A). Even after requiring T2W-hyperintensity to be in the correct infarct-related-artery territory (to reduce false positives post-hoc) prevalence was 59% (1-3 months), 32% (3-6 months), and 4% (>6-months). Individually, prevalence of MO (57%) and Increased-EDWT (45%) was low for <1-week-old infarcts but substantially increased in combination (77%, p<0.001) while retaining low prevalence for intermediate-aged infarcts (Figure 1, Panel B). Defining acute and chronic MI as <1 and ≥1-month-old, T2W sensitivity and specificity were 88% and 66%. For combined DE/Cine-CMR this was 74% and 97%. When removing patients with 1-6-month-old infarcts, T2W-CMR specificity increased to 83% (p<0.01). One-third of T2W-images were graded poor, nearly 5-fold higher than cine or DE-CMR (Figure 1, Panel C), as 52% and 92% of T2W-images had some myocardial signal drop-out (2.4±1.8 segments of 17-segments) and/or slow flow (7±3.5 segments).

## Conclusions

Sensitivity of T2W-CMR to detect <1-month-old infarcts is moderately high, but because edema may persist, T2W-CMR is less specific when including intermediate-aged infarcts (1-6 months). Although mildly less sensitive, the presence of MO or increased-EDWT on DE/cine-CMR is very specific, and unlike T2W-CMR image quality is rarely poor.

